# Clinic Time Required for Remote and In-Person Management of Patients With Cardiac Devices: Time and Motion Workflow Evaluation

**DOI:** 10.2196/27720

**Published:** 2021-10-15

**Authors:** Amber Seiler, Eliana Biundo, Marco Di Bacco, Sarah Rosemas, Emmanuelle Nicolle, David Lanctin, Juliette Hennion, Mirko de Melis, Laura Van Heel

**Affiliations:** 1 Cone Health Medical Group Greensboro, NC United States; 2 Deloitte Brussels Belgium; 3 Medtronic Mounds View, MN United States; 4 Centracare St. Cloud, MN United States

**Keywords:** cardiac implantable electronic devices, remote monitoring, patient management, clinic efficiency, digital health, mobile phone

## Abstract

**Background:**

The number of patients with cardiac implantable electronic device (CIED) is increasing, creating a substantial workload for device clinics.

**Objective:**

This study aims to characterize the workflow and quantify clinic staff time requirements for managing patients with CIEDs.

**Methods:**

A time and motion workflow evaluation was performed in 11 US and European CIEDs clinics. Workflow tasks were repeatedly timed during 1 business week of observation at each clinic; these observations included all device models and manufacturers. The mean cumulative staff time required to review a remote device transmission and an in-person clinic visit were calculated, including all necessary clinical and administrative tasks. The annual staff time to manage a patient with a CIED was modeled using CIED transmission volumes, clinical guidelines, and the published literature.

**Results:**

A total of 276 in-person clinic visits and 2173 remote monitoring activities were observed. Mean staff time required per remote transmission ranged from 9.4 to 13.5 minutes for therapeutic devices (pacemaker, implantable cardioverter-defibrillator, and cardiac resynchronization therapy) and from 11.3 to 12.9 minutes for diagnostic devices such as insertable cardiac monitors (ICMs). Mean staff time per in-person visit ranged from 37.8 to 51.0 and from 39.9 to 45.8 minutes for therapeutic devices and ICMs, respectively. Including all remote and in-person follow-ups, the estimated annual time to manage a patient with a CIED ranged from 1.6 to 2.4 hours for therapeutic devices and from 7.7 to 9.3 hours for ICMs.

**Conclusions:**

The CIED patient management workflow is complex and requires significant staff time. Understanding process steps and time requirements informs the implementation of efficiency improvements, including remote solutions. Future research should examine heterogeneity in patient management processes to identify the most efficient workflow.

## Introduction

### Background

The number of patients receiving and living with cardiac implantable electronic devices (CIEDs), including permanent pacemakers, implantable cardioverter-defibrillators (ICD), cardiac resynchronization therapy (CRT) devices, and insertable cardiac monitors (ICMs), has increased significantly in the past several years [1-3]. Accordingly, the burden for device clinics to manage follow-up visits has increased. Such follow-up visits, consisting of device interrogation and subsequent care changes (ie, reprogramming device settings and acting upon clinical findings), have traditionally been performed in person. Their frequency, essentially based on clinical guidelines, may vary depending on the facility, physician and patient preferences, and available resources [4]. As an alternative or complement to in-person visits, remote monitoring (RM) has become a guideline-recommended method for managing patients with CIEDs [5,6]. RM capabilities are a standard feature of modern CIEDs, and data are continuously transmitted through landlines or mobile networks, which supplies health care providers with critical clinical (eg, arrhythmias) and device-related (eg, battery longevity) information that allows them to adjust and optimize patient treatment accordingly. In the 2015 Heart Rhythm Society (HRS) Consensus Statement on remote interrogation and monitoring for CIEDs, endorsed by European Heart Rhythm Association and other international societies, RM combined with an annual in-person visit is recommended rather than in-person evaluation alone, with the strongest (class I) recommendation and the highest level of evidence (A) [5]. This recommendation is primarily because of earlier detection of clinical events, including atrial fibrillation, ventricular arrhythmias, and pause arrhythmias, to which RM enables faster clinical response and appropriate medical action [7-9]. Several studies have confirmed the clinical and economic benefits of RM, including improved patient outcomes and reduced health care use [5,9-13].

Furthermore, the literature has shown that the review of an RM transmission requires less staff time than an in-office interrogation, and RM is associated with greater patient adherence to device follow-up checks and a reduction in scheduled, often nonactionable in-office visits [10,14,15]. However, implementation in clinical practice of an overall management process for patients with CIEDs, incorporating both RM and in-person visits, can be challenging because of the scarcity of information on organizational models and requirements. The specific steps involved and the health care professional time required for these activities are poorly understood, which may hinder the implementation of optimal follow-up strategies, including remote solutions.

### Objective

This study aims to characterize the workflow processes and clinic staff time required for remote and in-person device follow-up of patients with CIEDs.

## Methods

### Data Collection

A time and motion workflow evaluation was performed in 11 CIED clinics internationally to characterize the discrete activities and associated time required for all tasks related to managing patients with CIEDs. Among the participating clinics, 6 were located in the United States, and 5 were located in Europe (3 in the United Kingdom, 1 in France, and 1 in Germany). Participating clinics were actively managing an average of 5758 (range: 870-22,000) patients with CIEDs, an average of 4217 patients in the United States and 7606 in Europe. All 11 clinics used guideline-recommended RM in combination with in-person device follow-up. Half (3) of the US clinics were located within academic institutions, and 3 of the 5 European clinics were academic.

A third-party observer prospectively collected data for one business week (5 days) at each clinic, recorded the tasks performed by the staff, and measured each task's duration with a stopwatch. Workflow measurements included all CIED types (permanent pacemaker, ICD, CRT, ICM) across any device manufacturer found within the clinic during the study week (Abbott, Biotronik, Boston Scientific, Medtronic, and Microport).

The observations included all activities related to managing patients with CIEDs and were categorized into 3 groups of activities: in-person clinic visits, remote transmission review, and other patient management activities not attributable to a specific patient device check (eg, patient triage and scheduling, identifying patients lost to follow-up, and telephone communication with patients). Owing to insufficient data collection on remote transmission review workflow activities at the German site, these observations were excluded from the analysis.

### Statistical Analysis

#### Staff Time Per Device Check for Remote and In-Person Device Follow-ups

Multimedia Appendix 1 lists all observed workflow steps occurring within each activity category (in-person clinic visit, remote transmission review, and other patient management activities). The differences in observed steps between categories are because of differences in device check scope; for example, assessing patient vitals or reprogramming device therapy are specific to in-person clinic visits. These lists are comprehensive of all possible steps observed, but all steps may not have occurred during each device check or in sequence as listed, as practices and workflow vary widely. Thus, to quantify the workload associated with an *average* patient device check (remote or in-person), the unit steps were weighted based on each step's likelihood of occurring in a given device check. The weighting factors are listed in Multimedia Appendix 2 [5,14,16-21] and were based on study observations where possible, supplemented by data from the literature. The mean time per remote and in-person device checks was calculated, including all clinical tasks and any administrative tasks related to that device check (eg, software access, documentation, scheduling follow-up, and sending information for billing).

For remote transmission review tasks, consideration of the transmission type and review process enabled further analyses. Transmissions were classified as nonactionable versus actionable (ie, requiring clinical follow-up because of either abnormal device functioning or a clinical patient event, such as an arrhythmia). It was assumed that 27% of transmissions would be actionable, based on a previously published time and motion evaluation [14]. Scenario analyses were performed to test the sensitivity of this parameter using additional literature-derived estimates [14,22,23]. We also estimated staff time spent on first- and second-line review of remote transmission data, considering that transmissions sometimes require escalation to more experienced staff for review and clinical decision-making. We assumed that 8.2% of transmissions would be sent for second-line review based on the aforementioned time and motion study [14]. For in-person clinic visits, 21.8% (27/124) of the visits were actionable based on study observations.

#### Annual Staff Time Per Patient for Remote and In-Person Device Follow-ups

On the basis of the calculated mean time per activity category, the annual staff time required to manage each patient with a CIED was modeled. The volume of remote transmissions per patient per year was based on real-world device transmissions from the calendar year 2016-2017 and included routine and alert-driven transmissions (Multimedia Appendix 2). For in-person clinical visits, it was assumed that each patient would have a routine visit per year according to clinical guidelines [6] and a number of unscheduled visits (ie, device alert or symptom-driven) based on the frequencies reported in the remotely monitored arm of published RM trials for each CIED type [16-20].

#### Annual Staff Time for Other Patient Management Activities

The annual time spent on other patient management activities not attributable to a specific patient device check was calculated based on observed clinic norms for performing tasks (eg, weekly identification of patients with disconnected monitors), whereas the frequency of telephone calls between the clinic and patient was based on a previous workflow study [21]. The per-patient workflow for in-person clinic visits, remote transmission review, and other patient management activities were extrapolated to the clinic level based on the average size of the clinics participating in the study (5758 patients).

#### Predictors of Clinic Efficiency

Prespecified subanalyses were performed to identify efficient clinical practices. Leveraging the same approach described above for modeling staff time per remote and in-person device check, the staff time per device check was modeled separately for the 3 US clinics in which vendor-neutral CIED management software (Medtronic Paceart Optima) was used during on-site observations, in comparison with 3 US clinics without management software. Similarly, the staff time per in-person visit was modeled separately for observations in which a tablet programmer was used versus visits in which a tablet programmer was not used.

#### Ethical Considerations

As this study was a workflow process evaluation that collected no patient or clinical data and only collected staff time measurements, the study protocol did not require approval from a local ethics committee or institutional review board. This study adhered to the General Data Protection Regulation and Health Insurance Portability and Accountability Act data privacy guidelines in Europe and the United States, respectively. The included sites consented to participate in the data collection process in accordance with their privacy requirements. Participating sites were required to have more than one employee of any given type (eg, nurse, physiologist, and physician) to preserve employee privacy, with all workflow data pooled across a staff of the same type.

## Results

### Data Collection

A total of 54 distinct workflow steps were observed and timed during the management of patients with CIEDs: 31% (17/54) for remote transmission review, 39% (21/54) for in-person clinic visits, and 30% (16/54) for other patient management activities such as patient phone calls and patient triage. The average time associated with each step is reported in Multimedia Appendices 3-5. During 11 total business weeks of data collection, observations included 276 in-person clinic visits (124/276, 44.9% the United States and 152/276, 55.1%, Europe), 1948 (1269/1948, 65.14% the United States and 679/1948, 34.86% Europe) individual remote transmission review tasks (not every step could be observed for each given transmission, as they often did not occur sequentially), and 440 other patient management tasks (the United States only). Considering all individual time recordings, approximately 50.21% (2424/4828) of the observations were in patients using pacemakers, 17.13% (827/4828) were in patients using ICD, 20.89% (1009/4828) were in patients using CRT, and 11.76% (568/4828) were in patients using ICM.

### Staff Time per Device Check for Remote and In-Person Device Follow-ups

Mean cumulative staff time required to review a remote device transmission ranged from 9.4 to 13.5 minutes (16.1-21.7 minutes for actionable and 6.1-11.2 minutes for nonactionable transmissions) for therapeutic devices (pacemaker, ICD, or CRT) and 11.3 to 12.9 minutes (17.3-20.3 minutes for actionable and 8.0-11.3 minutes for nonactionable transmissions) for ICMs.

Participating clinics generally used a two-level transmission-review process. A nurse or device technician performed a preliminary review (first-line review) to determine if the transmission requires the intervention of an advanced practitioner (second-line review performed by a nurse practitioner, physician assistant, or medical doctor). The staff time for a first-line review ranged from 11.9 to 13.0 minutes in the United States and 9.63 to 11.1 minutes in Europe, depending on device type. A second-line review ranged from 7.2 to 7.9 minutes in US clinics and 6.72 to 9.23 minutes in European clinics (Table 1).

Cumulative staff time per in-person clinic visit ranged from 37.8 to 51.0 minutes and 39.9 to 45.8 minutes for therapeutic devices and ICM, respectively (Table 1). For both remote transmission review and in-person clinic visits, the overall percentage of labor performed by each staff type (nurses, technician or medical assistants, medical doctors, physician assistants, or nurse practitioners, administrative assistants, and physiologists) is characterized by country in Table 2. Furthermore, the staff performing administrative tasks differed by site and region. In the United States, 46.3% (348/751) of all administrative workflow observations were performed by medical and administrative assistants, whereas 53.7% (403/751) of administrative tasks were performed by clinical practitioners. In the United Kingdom, all administrative workflow observations (n=165) were performed by clinical staff, including nurses, physiologists, and other advanced practitioners (Table 2).

Given that the estimated time to review an average remote transmission was dependent on the likelihood of a transmission being actionable, a series of scenario analyses were performed to test the sensitivity of this parameter. On the basis of the range of available literature-derived estimates (ranging from 8% to 27%), the time to review a transmission in the United States ranged from 11.9 to 13.5 minutes for patients with pacemakers, 10.8 to 12.7 minutes for patients with ICDs, 10.8 to 11.9 minutes for patients with CRTs, and 11.7 to 12.9 minutes for patients with ICMs. In Europe, the time ranged from 7.7 to 9.4 minutes for patients with pacemakers, 9.8 to 11.6 minutes for patients with ICDs, 10.7 to 12.4 minutes for patients with CRTs, and 9.6 to 11.3 minutes for patients with ICM.

**Table 1:** Mean cumulative staff time required per remote transmission review and in-person clinic visit.

Workflow activity					United States													Europe									
					PM^a^			ICD^b^			CRT^c^			ICM^d^			PM				ICD			CRT			ICM
**Remote device transmission review**																											
	Staff time per average transmission^e^, minutes			13.5			12.7			11.9			12.9			9.4				11.6			12.4			11.3	
	Number of transmissions per year (both scheduled and unscheduled transmissions)^f^			3.7			4.6			5.1			38.9			3.6				5.1			5.9			35.6	
Annual staff time for remote transmissions per patient, hours					0.8			1.0			1.2			8.4			0.6				1.0			1.2			6.7
**In-person clinic visits**																											
	Staff time per visit, minutes			50.1			51.0			43.4			45.8			41.2				37.8			40.9			39.9	
	Number of visits per year (both routine and event-driven visits)			1.5			1.7			1.7			1.3			1.5				1.7			1.7			1.3	
Annual staff time for clinic visits per patient, hours					1.3			1.4			1.2			1.0			1.0				1.1			1.1			1.0
Total annual per patient staff time, hours					2.1			2.4			2.4			9.3			1.6				2.0			2.4			7.7
**Type of remote device transmission**																											
	**Staff time required to review actionable versus nonactionable transmissions, minutes**																										
		Staff time per actionable transmission	19.8			20.1			16.1			17.3			18.3				20.6			21.7			20.3		
		Staff time per nonactionable transmission	11.2			9.9			10.3			11.3			6.1				8.3			9.0			8.0		
	**Distribution of staff time for first-line versus second-line review of remote transmissions, minutes**																										
		Staff time for first-line transmission review (relevant for all transmissions)	13.0			12.2			11.9			12.5			9.6				10.4			11.1			10.7		
		Staff time for second-line transmission review (required for only 8.2% of transmissions)	7.8			7.9			7.2			7.8			8.0				6.7			9.2			8.0		

^a^PM: permanent pacemaker.

^b^ICD: implantable cardioverter-defibrillator.

^c^CRT: cardiac resynchronization therapy.

^d^ICM: insertable cardiac monitors.

^e^The time required for an average transmission was modeled based on the assumption that 27% of transmissions are actionable and 73% of transmissions are nonactionable [14].

^f^The transmission volume is based on real-world data, and generalizability to other clinics will vary significantly depending on device programming practices, patient indications, and patient education.

**Table 2:** Percentage of cardiac implantable electronic devices management workload by staff type and region.^a^

Staff type and region		Nurse, n	Technician or medical assistant, n	Medical doctor, physician assistant, or nurse practitioner, n	Administrative assistant, n	Physiologist^b^, n
**In-person clinic visits**						
	United States	24	29	45	2	0
	United Kingdom	2	0	46	0	52
**Remote transmission review**						
	United States	53	10	35	3	0
	United Kingdom	0	0	0	0	100
**Other patient management (eg, calls and connectivity troubleshooting)^c^**						
	United States	48	51	0	1	0
**Time contribution by staff type: overall**						
	United States	44	20	34	2	0
	United Kingdom	1	0	19	0	80

**^a^**Labor share was calculated in the United States and the United Kingdom due to having multiple clinics observed in each country (6 and 3, respectively). As only one clinic was observed in Germany and France, there were insufficient data to perform this analysis in these countries.

^b^Clinical cardiac physiologists in the United Kingdom carry out procedures and investigations on patients related to diagnosis, monitoring, and treatment.

^c^The Other Patient Management data were only collected in the United States.

### Annual Staff Time Per Patient for Remote and In-Person Device Follow-ups

The mean number of transmissions per year per patient (including scheduled and unscheduled transmissions) ranged from 3.6 to 5.9 for therapeutic devices and 35.6 to 38.9 for ICMs (Table 1). In contrast, the number of expected in-person clinic visits per year ranged from 1.3 to 1.7 per patient. Although we seek to model time for an average clinic, the frequency of in-person and remote device checks will vary significantly between clinics depending on device programming practices, patient indications, and patient education.

Multiplying the staff time for each expected remote and in-person device check by the annual frequencies of device checks per year yielded an estimated total annual staff time of 1.6 to 2.4 hours to manage a patient with a therapeutic device and 7.7 to 9.3 hours for a patient with an ICM (Table 1). The higher staff time to manage a patient with an ICM was attributed to the increased transmission volume observed.

### Annual Staff Time for Other Patient Management Activities

The staff time required for other patient management tasks such as calling patients, troubleshooting device connectivity issues, identifying loss to follow-up, and triaging patients or transmissions (full task list provided in Multimedia Appendix 5) was estimated to be 17.3 minutes per patient annually. At the clinic level (based on the average 5758-patient clinic size of participating clinics), this translates to 1659.2 hours of staff time per year (31.9 hours per week).

### Predictors of Clinic Efficiency

A series of prespecified subanalyses were conducted to identify the predictors of clinic efficiency.

#### Vendor-Neutral Patient Management Software

The staff time required per remote and in-person device check was modeled separately for the 3 US clinics in which vendor-neutral CIED management software (Medtronic Paceart Optima) was used during on-site observations in comparison with the 3 US clinics without management software. On average, the total staff time to review a remote transmission was 2.1 minutes lower at sites with management software (11.5 vs 13.6 minutes). The staff time associated with an in-clinic visit was 2.2 minutes lower at clinics with management software (50.4 vs 52.6 minutes). For both remote transmission review and in-person clinic visits, the time savings were driven by the steps involved in electronic health record documentation. When extrapolated to an average clinic size of 5758 patients, the use of such software was associated with an estimated 10.1 cumulative staff hours saved during a clinic day (50.7 hours per week) based on 171 weekly clinic visits and 1335 weekly remote transmissions. Annually, this translates to 2639 hours of staff time saved, equivalent to 1.4 annual full-time equivalents.

#### Tablet-Based Programmers

In-person clinic visit staff time was modeled separately for visits in which a tablet-based CIED programmer was used (n=599 total observations) compared with visits in which a legacy programmer—which is large and cumbersome—was used (n=794). A tablet-based programmer was associated with an average of 5.2 minutes lower staff time (54 vs 49 minutes; 9.6% reduction) for a clinic visit, driven by reduced time for programmer device transport to a patient room and improved data connectivity with the electronic health record.

## Discussion

### Principal Findings

This study characterized the staffing resources necessary for cardiac device clinics to manage patients with CIEDs, including detailed time associated with each workflow step and breakdowns by device, geographic region, and staffing types. Although differences were observed across device types and geographic regions, the overall workload was found to be consistently substantial, regardless of CIED type and region.

As CIED technology advances, so do device data capabilities to inform and optimize patient care. The benefits of RM have been illustrated in several clinical studies, including faster event detection, improved patient outcomes, and reduced health care use. However, data alone will not result in clinical and economic benefits unless timely clinical action is taken. Clinical workflows must be optimized to capture the value of the device data.

The HRS consensus statement on RM outlined the importance of implementing a streamlined organization with clear roles and responsibilities to manage RM data in parallel with in-person follow-ups [5]. However, there is limited literature on how patients with CIEDs are managed in practice, including the workflow steps and the staffing requirements associated with each task, creating implementation challenges for new RM users. Protocols for remote management of patients with CIEDs have been developed, including the HomeGuide registry study [24], which implemented a dedicated nurse-physician team strategy. Similar to most sites in this study, a two-tiered remote transmission review structure was leveraged, in which nurses (or other similar practitioners) performed the initial transmission review and escalated critical events to a physician. However, this model may not apply to all device clinics, depending on the size, RM infrastructure, and staff resources available.

Although different organizational models, staff types, and workflows may exist in practice depending on the setting and available resources, the essential tasks required to manage patients with CIEDs remain similar. This evaluation sets a baseline by describing the essential activities performed by clinical staff to manage a population of patients with CIEDs and the time required to execute it. It also underscores the complexity of the current management of patients with CIEDs, identifying 54 distinct workflow tasks across three categories (remote transmission review, in-person clinic visits, and other patient management activities, such as patient phone calls and triaging).

Mean staff time required per remote transmission review and in-person clinic visit ranged from 9.4 to 13.5 minutes and 37.8 to 51.0 minutes, respectively, depending on device type. This validates previous research demonstrating the efficiency opportunities for RM [14]. The annual time per patient required for in-person device checks was relatively consistent across device types (1.0-1.4 hours), perhaps because of the low frequency of office visits required for patients being continuously monitored with RM. The annual time per patient for RM was higher in patients with diagnostic devices (ICMs: 6.7-8.4 hours per year vs only 0.6-1.2 hours for therapeutic devices) because of increased device transmissions both for routine data review and programmable automatic device alerts. However, it should be noted that the magnitude of the device transmission frequency with ICMs is highly dependent on the alert programming settings, patient indications, and patient education. The overall annual time required to follow and manage a patient with a CIED is lower for a patient with a therapeutic device (1.6-2.4 hours) than a patient with a diagnostic device (ICM: 7.7-9.3 hours).

As revealed by the predictors of efficiency analysis described above, even small improvements in the efficiency of CIED clinics can have a significant positive impact on time savings. Although our study observed meaningful time savings associated with the use of patient management software and tablet-based programmers, further research is needed to identify other strategies for optimal patient follow-up. For instance, the growing number of technologies capable of transmitting patient data to CIED clinics represents a challenge for data management [25,26]. Further innovation on solutions for integrating and storing data from this multitude of sources could enhance the efficient management of patients with CIEDs. This software also presents an opportunity for closer clinical care and patient safety; previous studies using the triaging and analytic capabilities of the PaceArt Optima system showed an improved enrollment of patients in RM [27] and identification and successful interventions for patients with suboptimal ICD programming [28] and low CRT pacing [29,30].

Our study observed a significant workload associated with fielding patient calls and troubleshooting connectivity, which is consistent with a recent study that found that more than 40% of patient calls received by CIED clinics pertain to troubleshooting RM equipment and transmission status [21]. Strategies to improve device connectivity—for instance, Bluetooth-enabled, smartphone-paired, or widespread use of other wireless monitors—could alleviate this significant workload burden on CIED clinics. Furthermore, optimizing the appropriate staffing types for each activity (eg, administrative tasks performed by administrative staff) could help clinics balance operational costs and resource availability.

A number of studies have previously estimated the time to perform remote and in-person device checks, yielding a wide range of time estimates, suggesting that there may be significant clinic-to-clinic variability [14,31-33]. To our knowledge, this study is the first multicenter, multinational study to describe comprehensive work requirements for managing patients with CIEDs. Considering the significant time-consuming activities related to follow up patients with CIEDs, appropriate funding needs to be in place to ensure that this crucial part of the patient care pathway is not overlooked. As RM is considered a guideline-recommended standard of care for all patients with CIEDs, hospital or clinic budget holders, payers, and reimbursement authorities should financially support its implementation and day-to-day practice. Funding and reimbursement of RM are variable and remain a challenge in many geographies today, as all stakeholders involved in this continuous service provision are often not remunerated or insufficient. Such a barrier affects RM adoption and its implementation as a standard of care. For this time and motion evaluation, countries where RM reimbursement is available were selected to avoid the influence of this lack of financial incentives on patient management organizations, which could be reflected in time measures. However, local reimbursement challenges persist, including limitations on specific cardiac devices, settings, and health care professionals in France and Germany. RM may not be suitable for every patient for different reasons (technical, clinical, or patient preference) but should be proposed to all eligible patients, in line with the medical recommendations and considering the current environment [34,35]. With the COVID-19 pandemic, RM benefits have been reinforced, also underlining the need to establish an appropriate infrastructure to manage patients remotely, which requires human, time, and financial investment.

Although it is widely acknowledged today that RM is a valuable tool for optimal follow-up of patients with CIEDs, to achieve this objective, infrastructure investments are required, including equipment (eg, additional computer or monitors) and setting up a specific clinic workflow organization involving sufficient human resources. As this infrastructural investment might not be an option in all settings, outsourcing remote patient management to other clinics or third-party arrhythmia review services could be an alternative to in-house implementation, as has been shown previously [36].

### Limitations

This study had several limitations. Owing to the real-world observational nature of this analysis, study measurements were reliant on the workflow taking place during the data collection week and were not systematically controlled for patient or center characteristics. This study describes the workflow observed at 11 centers in the United States and Europe, but the generalizability of these observations to other centers with different device populations and staffing resources is unknown. However, this is the first attempt at characterizing cardiac device clinic workflow in full and provides a first step in filling the knowledge gap around patient management practices and resource requirements.

In addition, as the time and motion methodology was designed as a clinic-perspective workflow characterization and did not follow patients longitudinally, we were unable to measure patient clinical metrics, such as device connectivity success and patient adherence to follow-ups. Finally, extrapolations were made using externally published data (eg, proportion of device checks requiring second-line assessment) and HRS guidelines for patient follow-up, and these assumptions may not be generalizable to all clinics. A series of sensitivity analyses were performed to test the impact of our assumption on transmission actionability. Although device check-level and annual resource use will be dependent on individual workflow practices, individual workflow step measurements can readily be used to create a workflow framework that is highly customizable to individual centers and circumstances.

### Conclusions

This observational study confirmed the complexity of the management of patients with CIEDs. The associated workflows require significant clinical and administrative staff time across in-person clinic visits, remote transmission review, and other patient management tasks. RM is an efficient component of managing patients with CIEDs, allowing for continuous follow-up of patients with reduced staff time required per device check. Detailed recommendations on organizational models for managing patients with CIEDs are warranted to ensure homogeneous follow-up, support RM implementation, and enable optimal patient care.

## Appendix

Multimedia Appendix 1 Workflow steps observed during the management of cardiac implantable electronic devices patients.

Multimedia Appendix 2 Modeling inputs.

Multimedia Appendix 3 Mean staff time required per instance for remote transmission review steps.

Multimedia Appendix 4 Mean staff time required per instance for in-person clinic visit steps.

Multimedia Appendix 5 Mean staff time required per instance for other patient management activities.

## Abbreviations

CIED: cardiac implantable electronic device
CRT: cardiac resynchronization therapy
HRS: Heart Rhythm Society
ICD: implantable cardioverter-defibrillator
ICM: insertable cardiac monitor
RM: remote monitoring
