# Supplementary material for: Clinic Time Required for Remote and In-Person Management of Patients With Cardiac Devices: Time and Motion Workflow Evaluation
Source: JMIR Cardio. 2021 Oct 15;5(2):e27720. doi: 10.2196/27720 (PMC8556635; doi:10.2196/27720)
Supplement: Multimedia Appendix 1 [file cardio_v5i2e27720_app1.docx]

Multimedia Appendix 1. Workflow steps observed during the management of CIED patients

| In-person Clinic Visit Activities |  | Remote Transmission Review Activities |  | Other Patient Management Activities |
| --- | --- | --- | --- | --- |
| *Diagnostic tasks:* |  | ***Diagnostic tasks:*** |  | ***Patient triaging:*** |
| Take vitals and history |  | Initial review of transmission |  | Employee accesses software |
| Device Interrogation |  | Advanced Practitioner reviews transmission |  | Check scheduling system for scheduled patient visits |
| Manual Threshold test/sensing |  | Review report for follow-up action |  | Identify patients with remote device alerts |
| Initial results review |  | ***Medical actions taken:*** |  | Identify scheduled/routine transmissions for review |
| Advanced Practitioner Consultation and review results |  | Communication to patient care team |  | Identify patients with remote device events |
| Review Advanced Practitioner report for follow-up action |  | Contact patient regarding alert received |  | ***Identification of patients with device connectivity issues:*** |
| *Medical actions taken:* |  | Implement care change (e.g. change medication, order tests, etc.) as follow-up to remote transmission |  | Identify unscheduled transmissions |
| Re-Program device therapy in-person |  | ***Administrative, documentation, and logistical tasks:*** |  | Identify patients with missing appointments |
| Reprogram device alerts in-person |  | Access patient file on EHR |  | Identify patients without a monitor |
| Discuss results with the patient |  | Verify transmissions transferred from PaceArt |  | Identify disconnected monitors |
| Change in care (e.g. change medication, order tests, etc.) as follow-up to visit |  | Open transmission |  | Identify patients not transmitting |
| *Administrative and logistical tasks:* |  | Add notation/comments |  | ***Telephone calls with patients:*** |
| Access patient file on EHR |  | Send transmissions to Advanced Practitioner |  | Patient call regarding troubleshooting device connectivity |
| Bring programmer into the exam room |  | Sign off transmissions |  | Patient calls in to confirm device transmission status |
| Add notation/ comments to EHR |  | Finalize report |  | Patient call regarding device battery status or device functioning |
| Attach report to EHR |  | Attach report to EHR |  | Patient call regarding symptoms or concerns |
| Transfer data to PaceArt Optima^TM^ |  | Send for billing |  | Patient call regarding scheduling/re-scheduling |
| Sign off in-person visit |  |  |  | Contact patient to request a manual device transmission |
| Report results sent to EHR from PaceArt |  |  |  | Documentation of telephone call with the patient (applicable for all call types above) |
| Check out patient |  |  |  |  |
| Schedule follow-up visit |  |  |  |  |
| Generate reminder/next appt letter |  |  |  |  |
