# Supplementary material for: Clinic Time Required for Remote and In-Person Management of Patients With Cardiac Devices: Time and Motion Workflow Evaluation
Source: JMIR Cardio. 2021 Oct 15;5(2):e27720. doi: 10.2196/27720 (PMC8556635; doi:10.2196/27720)
Supplement: Multimedia Appendix 2 [file cardio_v5i2e27720_app2.docx]

Multimedia Appendix 2. Modeling Inputs

| **Assumption** | **Value** | **Source** |
| --- | --- | --- |
| **In-person Clinic Visits:** | | |
| % of Visits requiring consultation with advanced care practitioner (e.g., physician, advanced nurse) | 50% | Study observation |
| % of Visits with device therapy reprogramming performed (therapeutic devices only) | 19% | Study observation |
| % of Visits with device alert reprogramming performed | 13% | Study observation |
| % of Visits that are actionable (i.e., require follow-up action such as changing medication, ordering labs) | 22% | Study observation |
| # of Routine in-person clinic visits per year per patient | 1 visit | HRS Consensus Statement Slotwiner et al. 2015 [5] |
| # of Unscheduled (alert or symptom-driven) visits per year per patient | - PM=0.5 visits - ICD=0.7 visits - CRT=0.7 visits - ICM=0.3 visits | Mabo et al. 2012 [16], Calo 2013 [17], Guedon-Moreau 2013 [18], Heidbuchel 2015 [19], Boriani 2017 [20] |
| **Remote Transmission Review:** | | |
| % of Transmissions sent for 2^nd^ line review | 8.2% | Cronin et al. 2012 [14] |
| % of Remote device checks that are actionable (i.e., require follow-up action such as changing medication, ordering labs) | 27% | Cronin et al. 2012 [14] |
| # of Remote transmissions per year (including routine device checks and alert-driven transmissions): | - Pacemaker:U.S.=3.7, Europe=3.6 - ICD: U.S.=4.6, Europe=5.1 - CRT: U.S.=5.1, Europe=5.9 - ICM: U.S.=38.9, Europe=35.6 | Analysis of CIED transmission data, 2016-2017 (Data on file) |
| **Other Patient Management Activities:** | | |
| # of Phone calls per year per patient:   - Troubleshooting connectivity - Transmission status - Device therapy or battery life - Discuss symptoms - Scheduling | - 0.33/year - 0.43/year - 0.14/year - 0.16/year - 0.35/year | Van Heel et al. 2020 [21] |
