# Supplementary material for: Clinic Time Required for Remote and In-Person Management of Patients With Cardiac Devices: Time and Motion Workflow Evaluation
Source: JMIR Cardio. 2021 Oct 15;5(2):e27720. doi: 10.2196/27720 (PMC8556635; doi:10.2196/27720)
Supplement: Multimedia Appendix 3 [file cardio_v5i2e27720_app3.docx]

**Multimedia Appendix 3. Mean Staff Time Required Per Instance for Remote Transmission Review Steps**

| Step | United States Time (seconds) | Europe  Time (seconds) |
| --- | --- | --- |
| *Diagnostic tasks:* | | |
| Initial review of transmission | 123.1 | 85.4 |
| Advanced Practitioner reviews transmission | - | 177.3 |
| Review report for follow-up action | 75.4 | 139.0 |
| *Medical actions taken:* | | |
| Communication to patient care team | 78.4 | - |
| Contact patient regarding alert received | 255.2 | 260.3 |
| Implement care change (medication, order tests, etc) | 206.8 | 83.0 |
| *Administrative, documentation, and logistical tasks:* | | |
| Access patient file on EHR | 10.6 | 86.5 |
| Verify transmissions transferred from PaceArt | 18.5 | - |
| Open transmission | 13.0 | 34.8 |
| Add notation/comments | 144.4 | 143.2 |
| Send transmissions to Advanced Practitioner | 56.3 | - |
| Sign off transmissions | 8.6 | - |
| Finalize report | 102.8 | - |
| Attach report to EHR | 29.0 | 130.1 |
| Send for billing | 61.8 | - |
