# Supplementary material for: Clinic Time Required for Remote and In-Person Management of Patients With Cardiac Devices: Time and Motion Workflow Evaluation
Source: JMIR Cardio. 2021 Oct 15;5(2):e27720. doi: 10.2196/27720 (PMC8556635; doi:10.2196/27720)
Supplement: Multimedia Appendix 4 [file cardio_v5i2e27720_app4.docx]

**Multimedia Appendix 4. Mean Staff Time Required Per Instance for In-person Clinic Visit Steps**

| Step | United States Time (seconds) | Europe  Time (seconds)* |
| --- | --- | --- |
| *Diagnostic tasks:* |  |  |
| Take vitals and history | 272.6 | 89.7 |
| Device Interrogation | 243.0 | 125.4 |
| Manual Threshold test/sensing | 107.4 | 238.5 |
| Initial results review | 485.0 | 175.2 |
| Advanced Practitioner Consultation and review results | 558.8 | 247.9 |
| Review Advanced Practitioner report for follow-up action | 438.9 | - |
| *Medical actions taken:* |  |  |
| Re-Program device therapy in-person | 251.9 | 182.7 |
| Re-program device alerts in-person | 91.6 | 72.3 |
| Discuss results with the patient | - | 111.4 |
| Change in care (medication, order tests, etc) | 206.8 | 83.0 |
| *Administrative, documentation, and logistical tasks:* |  |  |
| Access patient file on EHR | 22.2 | 38.7 |
| Bring programmer into the exam room | 102.8 | 85.2 |
| Add notation / comments to EHR | 253.5 | 267.2 |
| Attach report to EHR | 66.5 | 50.6 |
| Transfer data to PaceArt Optima^TM^ | 41.3 | - |
| Sign off in-person visit / Send for billing | 73.5 | - |
| Report results sent to EHR from PaceArt | 32.8 | - |
| Check out patient | 37.9 | 45.2 |
| Schedule follow-up visit | 168.5 | 175.9 |
| Generate reminder/next appt letter | 111.8 | 94.0 |
