# Supplementary material for: Clinic Time Required for Remote and In-Person Management of Patients With Cardiac Devices: Time and Motion Workflow Evaluation
Source: JMIR Cardio. 2021 Oct 15;5(2):e27720. doi: 10.2196/27720 (PMC8556635; doi:10.2196/27720)
Supplement: Multimedia Appendix 5 [file cardio_v5i2e27720_app5.docx]

**Multimedia Appendix 5. Mean Staff Time Required Per Instance for Other Patient Management Activities:**

| Step | Time (seconds) |
| --- | --- |
| *Patient triage:* |  |
| Employee accesses software | 9.1 |
| Check hospital scheduling system for scheduled patients | 11.6 |
| Identify patients with remote device alerts | 31.7 |
| Identify scheduled/routine transmissions for review | 15.9 |
| Identify patients with remote device events | 31.8 |
| *Identification of patients with device connectivity issues:* |  |
| Identify unscheduled transmissions | 25.6 |
| Identify patients with missing appointments | 130.0 |
| Identify patients without a monitor | 315.0 |
| Identify disconnected monitors | 64.1 |
| Identify patients not transmitting | 63.6 |
| *Telephone calls with patients:* |  |
| Patient call regarding troubleshooting device connectivity | 310.0 |
| Patient calls in to confirm clinic receipt of device transmission | 136.3 |
| Patient call regarding device-related questions (battery status, device functioning, MRI or other compatibility questions) | 132.8 |
| Patient call regarding symptoms or concerns | 315.3 |
| Patient call regarding scheduling/re-scheduling | 168.5 |
| Contact patient to request a device transmission | 262.3 |
| Documentation of telephone call (applicable for all call types above) | 163.3 |
